# Using whole genome sequencing to characterize *Clostridioides difficile* isolates at a tertiary center in Melbourne, Australia

**DOI:** 10.1017/ash.2023.529

**Published:** 2024-01-12

**Authors:** Alice Liu, Eddie Chan, Victoria Madigan, Vivian Leung, Lucille Dosvaldo, Norelle Sherry, Benjamin Howden, Katherine Bond, Caroline Marshall

**Affiliations:** 1 Victorian Infectious Diseases Service, The Royal Melbourne Hospital, Melbourne, Victoria, Australia; 2 Microbiology Department, Royal Melbourne Hospital, Melbourne, Victoria, Australia; 3 Infectious Diseases Department, The Northern Hospital, Melbourne, Victoria, Australia; 4 Infection Prevention and Surveillance Service, Royal Melbourne Hospital, Melbourne, Victoria, Australia; 5 Microbiological Diagnostic Unit Public Health Laboratory, Department of Microbiology & Immunology, University of Melbourne at the Peter Doherty Institute for Infection & Immunity, Melbourne, Victoria, Australia; 6 Department of Infectious Diseases, University of Melbourne at the Peter Doherty Institute for Infection and Immunity, Melbourne, Victoria, Australia

## Abstract

**Objective:**

*Clostridioides difficile* infection (CDI) is the commonest cause of healthcare-associated diarrhea and undergoes standardized surveillance and mandatory reporting in most Australian states and territories. Historically attributed to nosocomial spread, local and international whole genome sequencing (WGS) data suggest varied sources of acquisition. This study describes *C. difficile* genotypes isolated at a tertiary center in Melbourne, Australia, their likely source of acquisition, and common risk factors.

**Design:**

Retrospective observational study.

**Setting:**

The Royal Melbourne Hospital (RMH), a 570-bed tertiary center in Victoria, Australia.

**Methods:**

Short-read whole genome sequencing was performed on 75 out of 137 *C. difficile* isolates obtained from 1/5/2021 to 28/2/2022 and compared to previous data from 8/11/2015 to 1/11/2016. Existing data from infection control surveillance and electronic medical records were used for epidemiological and risk factor analysis.

**Results:**

Eighty-five (62.1%) of the 137 cases were defined as healthcare-associated from epidemiological data. On genome sequencing, 33 different multi-locus sequence type (MLST) subtypes were identified, with changes in population structure compared to the 2015–16 period. Risk factors for CDI were present in 130 (94.9%) cases, including 108 (78.8%) on antibiotics, 86 (62.8%) on acid suppression therapy, and 25 (18.2) on chemotherapy.

**Conclusion:**

In both study periods, most *C. difficile* isolates were not closely related, suggesting varied sources of acquisition and that spread of *C. difficile* within the hospital was unlikely. Current infection control precautions may therefore warrant review. Underlying risk factors for CDI were common and may contribute to the proportion of healthcare-associated infections in the absence of proven hospital transmission.

## Background

*Clostridioides difficile* infection (CDI) is the most common cause of healthcare-associated diarrhea,^1^ leading to significant burden on patients and the healthcare system. In 2013, the Australian Commission for Safety and Quality in Healthcare (ACSQHC) developed a national approach to surveillance consistent with internationally endorsed definitions and recommendations.^2,3^ In Victoria, all public hospitals are required to report CDIs to the Victorian Healthcare-Associated Surveillance System (VICNISS) Coordinating Centre,^1^ which provides standardized surveillance for healthcare-associated infections across public and private health services in Victoria. For CDIs, this includes classification into (1) healthcare-associated, hospital onset, (2) healthcare-associated, community onset, and (3) community-acquired cases, using standardized definitions based on time from healthcare contact. These surveillance requirements focus on epidemiological data and do not routinely record genotypes that characterize different *C. difficile* isolates.

Historically, nosocomial spread has been considered the main source of CDI and forms the basis for recommended infection control strategies in the healthcare setting such as hand hygiene and contact precautions.^4^ However, using whole genome sequencing (WGS) technology, local and international data demonstrate varied sources of CDI acquisition and increasing prevalence of community-associated disease.^5–9^ These observations merit ongoing close surveillance of CDI prevalence in local settings and demonstrate the utility of WGS in reporting CDI data, along with identification of common risk factors for infection.

The Royal Melbourne Hospital (RMH) is a large university-affiliated tertiary referral hospital that provides services including trauma surgery, neurosurgery, renal transplantation, hematology, and bone marrow transplantation units. Local infection control policies for confirmed CDIs include the allocation to a single room with dedicated toileting facilities, use of contact precautions (gowns and gloves) and hand washing with soap and water if gloves are not worn (otherwise alcohol-based hand rub is recommended), as well as use of sporicidal disinfectant for cleaning.

A previous study at RMH in 2015–16 showed that there was genomic diversity among isolates and WGS demonstrated that many isolates previously thought to be related, based on epidemiology, PCR ribotyping, and/or MLST, were in fact not genetically related, suggesting little if any in-hospital transmission. Infection control interventions were not changed at that time. Recent review of CDI surveillance data at RMH has revealed an increase in incidence over the last 12 months. It is currently unclear if this observation is attributable to transmission between patients, growing community transmission, or other factors. Characterizing the epidemiology and genomics of the *C. difficile* isolates at our hospital during this period and comparing with previous observations will inform future infection prevention policies.

### Aims and objectives

The primary objectives of this study are to a) describe the genomic epidemiology of *C. difficile* isolated among inpatients at RMH between 1/5/2021 and 28/2/2022 using genomic analysis; b) classify cases into the following types of CDI using standardized surveillance definitions: (1) healthcare-associated, hospital onset, (2) healthcare-associated, community onset, and (3) community-acquired; and c) identify any hospital transmission events that may warrant changes to current infection control practices.

As secondary objectives, this project compares the genomic diversity of *C. difficile* isolated during the current study period to the previous study period 2015–16 and describes risk factors for CDI among patients from whom a positive *C. difficile* culture was obtained.

## Methods

This was a single-center, retrospective, observational analysis of existing microbiological and surveillance data using *C. difficile* isolates identified at RMH between 1/5/2021 and 28/2/2022, as reported to the VICNISS Coordinating Centre. CDI cases were classified using epidemiological data according to the following standardized definitions: (1) healthcare-associated, hospital onset: CDI with symptom onset >48 hours after hospital admission; (2) healthcare-associated, community onset: CDI with symptom onset within 48 hours of admission and within 4 weeks of discharge from a healthcare facility; and (3) community-acquired: CDI with symptom onset in the community or within 48 hours of admission, but >12 weeks after discharge from a healthcare facility. Cases that do not meet these criteria are classified as indeterminate.

At RMH, laboratory diagnosis of CDI is established through a combination of culture and polymerase chain reaction (PCR) methods, a process which has remained consistent since 2011. In brief, all unformed fecal specimens are collected for *C. difficile* testing. Specimens are cultured on chromID selective *C. difficile* agar (bioMerieux, Marcy L’Etoile, France) and incubated in anaerobic conditions for 20–24 hours. Positive cultures undergo matrix-assisted laser desorption/ionization-time of flight (MALDI-TOF) mass spectrometry to confirm the identification of *C. difficile*, followed by toxin B gene detection by PCR on GeneXpert (Cepheid, Sunnyvale, CA, USA).

For patients who had more than one episode of CDI during the study period, only the first isolate was included for analysis. Positive isolates that were available from storage were retrieved from the RMH Microbiology Laboratory or the Microbiological Diagnostic Unit Public Health Laboratory (MDU PHL) to undergo short-read Illumina WGS (as previously described),^10^ *in silico* multi-locus sequence typing (MLST) and core single-nucleotide polymorphism (SNP) phylogenetic analysis at MDU PHL according to NATA-accredited workflows (to ISO15189 standards). Briefly, single colonies from pure isolates were placed in lysis buffer and then underwent DNA extraction, library preparation (Nextera XT workflow), and short-read Illumina sequencing (NextSeq500 platform, San Diego, CA, USA). Reads and assemblies underwent standard quality control (QC); once passed, they were assembled using shovill (v1.0.4)^11^ and typed using the mlst tool (v2.19.0)^12^. For sequence types (STs) with >1 case, a core SNP phylogenetic analysis was performed from an alignment of all genomes from that ST (using snippy)^13^ and visualized using IQTree,^14^ to identify genomic clusters, using a cut-off of ≤2 SNPs. Microbiological data were collected and analyzed in accordance with National Pathology Accreditation Advisory Council (NPAAC) standards.

Where potential genomic clusters were identified, additional review of patient records and bed movements was conducted to establish potential epidemiological links.

For the secondary outcome analysis, results from previous WGS of *C. difficile* isolates were used as a comparator. Clinical data on CDIs were collected from 8/11/2015 to 1/11/2016, and isolates were collected for WGS from 1/5/2016 to 31/8/2016. Forty-six isolates were collected over the latter four-month period and underwent identical *C. difficile* diagnostic processes in the RMH laboratory as with the 2021–22 cohort, using the same workflows for sequencing and analysis (using an updated MLST database).

Medication prescribing and comorbidity data were collected via retrospective review of hospital electronic patient records. Presence of the following risk factors for CDI acquisition and/ or severe disease was included (within 2 months of CDI diagnosis): use of antibiotics, acid suppression therapy, chemotherapy, other immunosuppressive medications or surgery. Data were limited to inpatient and outpatient attendances at RMH; details on community prescribing and presentations to other health services were not routinely available. Patient bed and ward locations were collected from electronic patient records and the hospital patient administration system, iPM (iSoft, Sydney, Australia). Antibiotic susceptibility testing was carried out using Clinical & Laboratory Standards Institute (CLSI) laboratory methods. Epidemiological analysis was conducted using R version 4.1.2 (R Core Teams 2021).

## Results

### Primary outcome

A total of 150 episodes of CDI from 137 patients were confirmed by the laboratory during the study period. Excluding duplicates, 137 cases of CDI were included, of which 75 isolates were available for WGS (57 were not stored and five either failed to grow or had a species identification incongruous with *C. difficile* after sequencing). There were 85 (62.1%) designated as healthcare-associated isolates and 38 (27.7%) as community-acquired isolates. A positive *C. difficile* toxin B PCR was detected in 124 (90.5%) cases. The VICNISS classifications of all CDIs and CDIs that underwent WGS are summarized in Tables 1 and 2, respectively.

**Table 1.** VICNISS CDI categories: all cases

CDI category	No. of cases (%)	
	2015–16 (*N* = 140)	2021–22 (*N* = 137)
Healthcare-associated, hospital onset	52 (37.1)	52 (38.0)
Healthcare-associated, community onset	6 (4.3)	33 (24.1)
Community-acquired	55 (39.3)	38 (27.7)
Toxin negative	27 (19.3)	13 (9.5)
Incomplete data	0 (0.0)	1 (0.7)

**Table 2.** VICNISS CDI categories: cases that underwent WGS

CDI category	No. of cases (%)	
	2015–16 (*N* = 49)	2021–22 (*N* = 75)
Healthcare-associated, hospital onset	20 (40.8)	32 (42.7)
Healthcare-associated, community onset	4 (8.2)	17 (22.7)
Community-acquired	25 (51.0)	17 (22.7)
Toxin negative	0 (0.0)	9 (12.0)
Incomplete data	0 (0.0)	0 (0.0)

**Figure 1.**
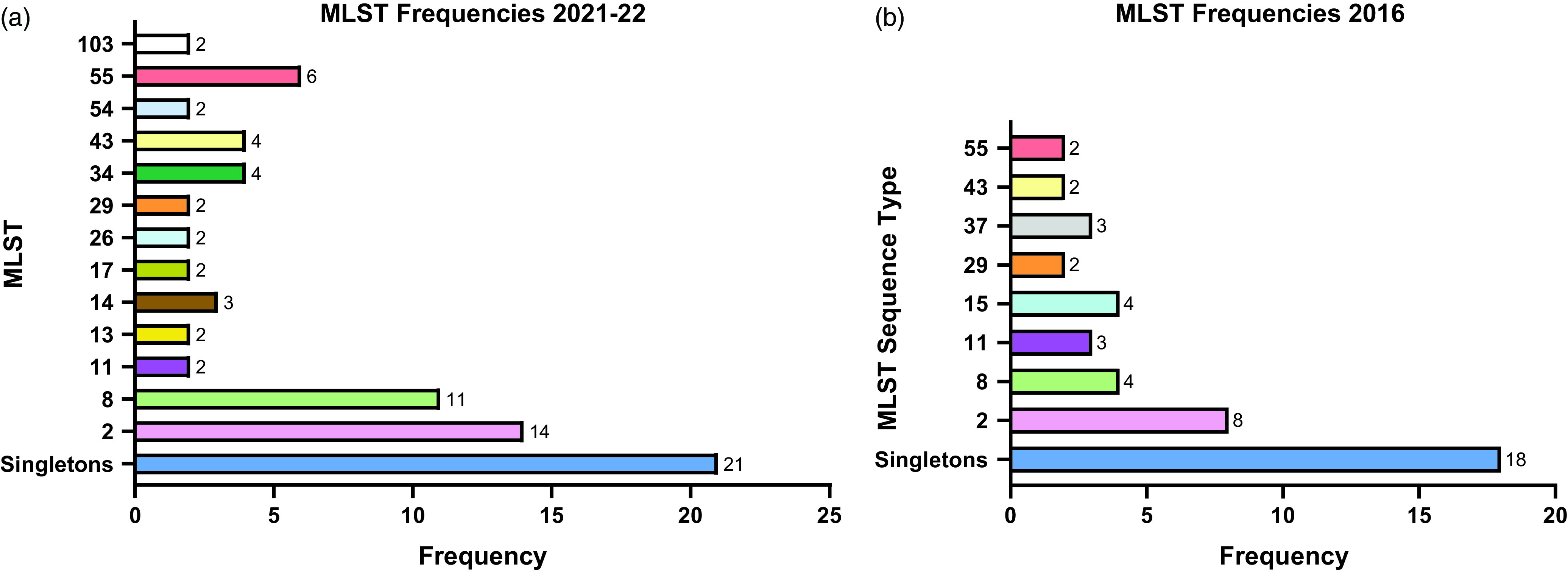
(a and b) MLST frequencies across both study periods. MLST: multi-locus sequence type. Sequence types isolated only once are designated as singletons.

Of the 75 isolates that underwent WGS, the sequences were polyclonal, covering 33 STs, including several sequence types isolated only once (“singletons”) and four novel STs (Figure 1a). A full list of STs is outlined in Supplementary Table 1.

Two potential clusters (patient pairs) were identified by phylogenetic analysis; the first including two isolates of ST8 collected from two patients (patients A and B), and the second including two isolates of ST103 collected from two different patients (patients C and D). In the ST8 cluster, patient A had two episodes of CDI during two separate admissions one month apart; only the second episode of CDI was contemporaneously linked to Patient B, each being diagnosed one day apart. Patients A and B were admitted on the same day, under different medical teams, to different wards with no overlapping bed movements prior to confirmation of their CDI. Patient A was tested the day after admission and patient B on the day of admission. Each patient developed symptoms at home prior to presentation and had received antibiotics within one month prior to admission. Both patients were on chemotherapy in the outpatient setting and had attended other outpatient hospital services in the 4 weeks prior to CDI diagnosis without overlap in location. In the ST103 cluster, patients C and D were each admitted under different surgical teams 3 weeks apart, to different wards on different floors with no overlapping bed movements during their inpatient stay. Patient C was diagnosed first, having developed symptoms on day 10 of their admission in the setting of recent surgery and post-operative antibiotic therapy. Patient D developed symptoms at home, 14 days after patient C’s diagnosis and in the setting of antibiotic use in the community. Overall, definite epidemiological links within either cluster could not be established.

### Secondary outcomes

In the earlier 2015–16 analysis, WGS was performed on 46 isolates collected between 1/5/2016 and 31/8/2016, with a population structure that differed in distribution and frequency of STs compared to the 2021–22 data set (Figure 1b). A full list of the 2016 STs is provided in Supplementary Table 2. Polyclonality was again observed during this study period, including the detection of novel sequences distinct from those identified in the latter study period. A broader range of STs were observed in the current 2021–22 group. In both study periods, STs 2, 8, 11, 29, 43, and 55 all had more than one isolate, and ST2 and 8 remained the most commonly detected STs.

Of the 46 isolates from the earlier study period that underwent WGS, four isolates from four different patients (patients A, B, C, and D) were found to be closely related in a single cluster on phylogenetic analysis. On further epidemiological review, there was one potential ward-based contact between patients A and B; patient A was admitted to the same ward as patient B one week after patient B, who was moved to a different ward on a different floor several hours later. Both patients A and B were diagnosed with CDI on the same date, 2 days after their putative ward contact. There was one potential floor-wide contact between patients A and C, who were admitted to different wards one week apart from each other and attended the same dialysis center in the hospital, but never on the same day. Patient C developed CDI 6 weeks after patient A. There was one possible hospital-wide contact between patient C and D. Patient C was admitted to the hospital on the same day as patient D, but there were no overlapping bed locations during their stays. Patient C was diagnosed with CDI on the same day that patient D was discharged, who developed CDI 9 days later. Definitive epidemiological links could not be established between patients A, C, and D. All four patients in the cluster had predisposing risk factors for the development of CDI.

Forty-nine episodes of CDI were investigated during the 2015–16 study period; 24 (49.0%) were classified as healthcare-associated, and 25 (51.0%) were community-acquired. *C. difficile* toxin B was detected in 35 (71.4%) cases.

The prevalence of risk factors for CDI acquisition and/ or severe disease is summarized in Tables 3 and 4. Data were available for 80 out of 140 episodes of CDI captured between 8/11/2015 and 1/11/2016.

**Table 3.** Prevalence of risk factors for CDI: all cases

Risk factor	2015–16	2021–22
	*n* (%), *N* = 80	*n* (%), *N* = 137
Any risk factor (within last 2 months)	74 (92.5)	130 (94.9)
Antibiotics	62 (77.5)	108 (78.8)
Acid suppression therapy	53 (66.3)	86 (62.8)
Chemotherapy	18 (22.5)	25 (18.2)
Other immunosuppression	14 (17.5)	48 (35.0)
Surgery	14 (17.5)	31 (22.6)

**Table 4.** Prevalence of risk factors for CDI: cases that underwent WGS

Risk factor	2015–16	2021–22
	*n* (%), *N* = 46	*n* (%), *N* = 75
Any risk factor (within last 2 months)	45 (97.8)	72 (96.0)
Antibiotics	38 (82.6)	68 (80.0)
Acid suppression therapy	34 (73.9)	43 (56.6)
Chemotherapy	12 (26.1)	14 (18.4)
Other immunosuppression	16 (34.8)	15 (19.7)
Surgery	9 (19.6)	12 (16.0)

## Discussion

While the genomic variation of CDI is well characterized in certain countries such as the United States and the UK,^7,15–17^ it has been less studied in the Australian context, where current literature focuses predominately on ribotyping methods.^6,7^ The traditional reference standard, ribotype sequencing is increasingly being replaced by WGS, which provide greater discriminatory power to the SNP level.^18,19^ During this transition period, ribotypes and their equivalent STs are often used together to compare data derived using the two different methods.

Recent WGS efforts at another Australian hospital indicated the plausible spread of non-toxigenic *C. difficile* ribotypes from diverse community sources to the healthcare setting.^20^ However, the absence of aggregated WGS data on Australian community and healthcare *C. difficile* isolates means it may be difficult to draw direct comparisons between the pathogenic STs identified in our cohort and community trends. Nevertheless, most isolates described in this study were not closely related based on genomic sequence analysis. These findings mirror the 2015–16 WGS investigation of *C. difficile* isolates at our hospital, which also demonstrated a strongly polyclonal population. We observed genetic diversity in our *C. difficile* isolates across both study periods, including the detection of different novel STs. This may reflect separate acquisition events from distinct sources such as environmental reservoirs, consistent with other larger WGS studies.^5^ Together, these findings imply that the vast majority of acquisition of *C. difficile* was highly unlikely due to hospital-associated transmission.

In the absence of linked CDI cases, the emphasis on current vertical prevention methods for transmission between patients may need to be revised. For example, the utility of contact precautions in certain cases of CDI has been interrogated in an earlier prospective analysis, which demonstrated low transmissibility of toxigenic, non-hypervirulent strains of *C. difficile* over a 10-year period after ceasing contact precautions for patients who were continent and had dedicated toileting facilities.^21^ Our findings will help inform ongoing infection control practices at our institution and elsewhere.

Overall, healthcare-associated CDIs (as designated by epidemiologic criteria) were more common in the current data set, with 85 (62.1%) identified during the 2021–22 study period. This is higher than the previous observation in 2015–16, but consistent with state-wide figures reported by VICNISS.^22^

Our phylogenetic analysis identified two patient pairs of potential hospital spread during the 2021–22 study period. Epidemiological review of these clusters did not reveal any overlapping patient bed locations or movements to indicate ward-based transmission, however definitive exclusion of other routes of transmission remains challenging. Given the polyclonal population structure observed in this analysis, which likely reflects some of the diversity of community infections and carriage, it is possible that these patients independently acquired their infections from a similar source in the community rather than acquisition in hospital.

Underlying risk factors for CDI were common across both study periods and proven hospital transmission. Antibiotic and acid suppression medication use were the most frequently identified risk factors, consistent with well-established evidence about their role in the pathogenesis of CDIs.^23–26^ Both agents contribute to dysbiosis of the gut microbiome, while acid suppression medications also elevate gastric pH; these mechanisms each reduce the barrier to colonization by *C. difficile* and increase the risk of clinical infection.^27^ Chemotherapy use was also common in our study and may increase the risk of CDI through direct damage to the intestinal mucosa, depletion of mucosal immune defenses, and significant modification of the gut microbiome.^28^

Our findings of diverse *C. difficile* STs and a high prevalence of predisposing risk factors are consistent with prior studies^29^ and may reflect community acquisition or carriage and emergence of healthcare-associated CDI due to exposure to predisposing medications in hospital. These risk factors are particularly relevant to the large population of immunocompromized patients cared for at our institution, many of whom receive broad-spectrum antimicrobials, proton-pump inhibitors in addition to their immunosuppression and may be at greater risk of mortality or morbidity due to CDI.^30^ Stewardship of predisposing medications and proactive identification of other risk factors present useful areas of focus in the prevention and clinical management of healthcare-associated CDI.

Our study has several limitations. Firstly, not all CDIs recorded during the study period had the corresponding *C. difficile* isolate stored; only a subset was available for WGS, which may not represent the full genomic variability of CDIs at our hospital. As a single-center study, the range and frequency of STs observed may not be generalizable to other centers. Furthermore, transmission between institutions (eg, due to inter-hospital transfer) was not captured in our epidemiological analysis, which only examined patient movements within our health service. Asymptomatic testing is not routine in our institution, therefore linked cases of asymptomatic *C. difficile* carriage may have been missed. Dedicated point prevalence studies may provide estimates of carriage for future reference.

There are some notable strengths to our study. We applied robust WGS techniques and phylogenetic analysis from an experienced and ISO-accredited reference laboratory, with the capacity for reproducible diagnostic methods for outbreaks and surveillance purposes, including comparison between healthcare centers. By comparing to the previous WGS analysis of isolates from 2015 to 16 conducted using the same techniques, we were also able to evaluate temporal trends in *C. difficile* STs and show that the putative epidemiology of CDI at our hospital has not altered in the approximately seven years between studies. This reaffirms our previous analysis to show minimal in-hospital transmission events and may support future evaluation of the benefits of *C. difficile*-specific infection control measures at our institution. Shifting focus from solely reducing transmission to preventing disease may also be justified.

## Conclusions

In conjunction with epidemiological tracing, WGS is a valuable tool in the evaluation of CDIs that may have utility in certain surveillance and outbreak investigation settings. Using a combination of these techniques, we describe a genomically diverse range of *C. difficile* isolates at an Australian tertiary center over a 7-year period, suggesting hospital transmission of CDI is an uncommon phenomenon. Given varied sources of *C. difficile* acquisition are likely, review of our infection control precautions may be worthwhile. Additionally, in the case of a suspected hospital outbreak, strong epidemiological links should be considered as well as WGS. Risk factors for CDI remained common and warrant ongoing attention to minimize resultant mortality and morbidity.

## Supporting information

Liu et al. supplementary material Liu et al. supplementary material

## Supplementary material

For supplementary material accompanying this paper visit http://doi.org/10.1017/ash.2023.529. click here to view supplementary material
