# Supplementary material for: Using whole genome sequencing to characterize Clostridioides difficile isolates at a tertiary center in Melbourne, Australia
Source: Antimicrob Steward Healthc Epidemiol. 2024 Jan 12;4(1):e7. doi: 10.1017/ash.2023.529 (PMC10789990; doi:10.1017/ash.2023.529)
Supplement: Liu et al. supplementary material [file S2732494X23005296sup001.docx]

**Supplementary Appendix**

Supplementary table 1: MLST sequence types, 2021-22

| STs with single isolates | | STs with >1 isolate | |
| --- | --- | --- | --- |
| MLST | n (%), N = 75 | MLST | n (%), N = 75 |
| 3 | 1 (1.3) | 2 | 14 (18.7) |
| 6 | 1 (1.3) | 8 | 11 (14.7) |
| 13 | 1 (1.3) | 11 | 2 (2.7) |
| 35 | 1 (1.3) | 14 | 3 (4.0) |
| 42 | 1 (1.3) | 17 | 2 (2.7) |
| 48 | 1 (1.3) | 26 | 2 (2.7) |
| 49 | 1 (1.3) | 29 | 2 (2.7) |
| 51 | 1 (1.3) | 34 | 4 (5.3) |
| 53 | 1 (1.3) | 43 | 4 (5.3) |
| 63 | 1 (1.3) | 54 | 2 (2.7) |
| 82 | 1 (1.3) | 55 | 6 (8.0) |
| 110 | 1 (1.3) | 103 | 2 (2.7) |
| 139 | 1 (1.3) |  |  |
| 234 | 1 (1.3) |  |  |
| 236 | 1 (1.3) |  |  |
| 237 | 1 (1.3) |  |  |
| 258 | 1 (1.3) |  |  |
| Novel 1 | 1 (1.3) |  |  |
| Novel 2 | 1 (1.3) |  |  |
| Novel 3 | 1 (1.3) |  |  |
| Novel 4 | 1 (1.3) |  |  |

Supplementary Table 2: MLST sequence types, 2015-16

| STs with single isolates | | STs with >1 isolate | |
| --- | --- | --- | --- |
| MLST | n (%), N = 46 | MLST | n (%), N = 46 |
| 3 | 1 (2.2) | 2 | 8 (17.4) |
| 6 | 1 (2.2) | 8 | 4 (8.7) |
| 9 | 1 (2.2) | 15 | 4 (8.7) |
| 10 | 1 (2.2) | 11 | 3 (6.5) |
| 13 | 1 (2.2) | 37 | 3 (6.5) |
| 14 | 1 (2.2) | 29 | 2 (4.3) |
| 17 | 1 (2.2) | 43 | 2 (4.3) |
| 26 | 1 (2.2) | 55 | 2 (4.3) |
| 34 | 1 (2.2) |  |  |
| 35 | 1 (2.2) |  |  |
| 39 | 1 (2.2) |  |  |
| 46 | 1 (2.2) |  |  |
| 49 | 1 (2.2) |  |  |
| 54 | 1 (2.2) |  |  |
| 388 | 1 (2.2) |  |  |
| Novel A | 1 (2.2) |  |  |
| Novel B | 1 (2.2) |  |  |
| Novel C | 1 (2.2) |  |  |
